# *Notes from the Field:* Domestically Acquired Verona Integron-Mediated Metallo-β-Lactamase-Producing Enterobacteriaceae — Indiana, 2016–2017

**DOI:** 10.15585/mmwr.mm6725a6

**Published:** 2018-06-29

**Authors:** D.J. Shannon, Sara Blosser, Maroya Walters, Alex Kallen, Christine Feaster

**Affiliations:** ^1^Indiana State Department of Health; ^2^Indiana State Department of Health Laboratories; ^3^Division of Healthcare Quality Promotion, CDC.

Beginning in January 2016, Verona integron-mediated metallo-β-lactamase (VIM) producing carbapenem-resistant Enterobacteriaceae (CRE) were identified in Indiana. CRE are an emerging antibiotic-resistant public health threat. CRE spread might be largely due to the emergence of carbapenemase-producing CRE (CP-CRE). Carbapenemases are generally encoded on mobile genetic elements that are easily transferred between bacterial strains, greatly increasing their potential for spread (*1**–**3*). Furthermore, CP-CRE pose a risk because of their extensive drug resistance, increased associated mortality, and national lack of public health laboratory capacity for detection prior to 2016 (*2**,**4**,**5*).

The geographic distribution of carbapenemases varies globally. In the United States, the carbapenemase most frequently identified among Enterobacteriaceae is *Klebsiella pneumoniae* carbapenemase; others are less common and are most often identified in patients who recently received health care outside the United States. For example, VIM is frequently identified in southern Europe and Southeast Asia; however, it is infrequently reported from the United States (*1*–*3*).

In December 2015, the Indiana State Department of Health (ISDH) mandated reporting of CP-CRE, allowing for statewide identification and response to CP-CRE. To facilitate this reporting, the ISDH laboratories hosted CP-CRE workshops in which clinical laboratorians were trained in the detection of carbapenemases via currently available phenotypic testing methods. The ISDH laboratories provide CP-CRE characterization in real time, allowing for timely public health intervention. Upon detection of CP-CRE, the ISDH provides education and technical assistance to health care facilities to ensure rapid implementation of proper infection control procedures. Each patient from whom a CP-CRE isolate is identified is investigated by the local health department to characterize demographics and CP-CRE risk factors, including recent health care exposures and international travel during the preceding 6 months.

During January 2016–December 2017, 649 CP-CRE isolates were reported across Indiana, including nine VIM-producing CP-CRE (VIM-CRE) isolates from seven patients. VIM was the most commonly identified carbapenemase after *Klebsiella pneumoniae* carbapenemase. Seven different species were identified from the nine VIM-producing isolates; one patient was found be colonized or infected with three different VIM-producing organisms over a 15-month period (Table). All seven patients had overnight stays in Indiana health care facilities, and none had documented international travel in the 6 months preceding specimen collection.

**TABLE:** Verona integron-mediated metallo-β-lactamase–producing carbapenem-resistant Enterobacteriaceae (N = 9) isolated from seven patients in health care facilities — Indiana, January 1, 2016–December 31, 2017

Patient	Age (yrs)	Sex	Specimen collection date	Specimen	Organism	Health care exposure history in 6 months preceding specimen collection	Antibiotic use in 6 months preceding specimen collection	Other resistant organisms identified in 6 months preceding specimen collection
1*	36	M	01/19/2016	Wound	*Proteus mirabilis*	ACH	Unknown	CRE, MDR-AB, ESBL
			01/27/2017	Urine	*Klebsiella pneumoniae*	ACH	Yes	CP-CRE, MDR-AB, MRSA
			03/24/2017	Urine	*Providencia rettgeri*	ACH	Yes	CP-CRE, MDR-AB, MRSA
2	28	M	03/21/2016	Wound	*Enterobacter cloacae* complex	ACH	Yes	MRSA
3	67	M	10/01/2016	BAL	*Enterobacter cloacae* complex	ACH, LTACH, LTCF	Yes	CRE, MRSA, MDR-PA, CDI
4	94	F	12/12/2016	Urine	*Klebsiella pneumoniae*	LTCF	Yes	VRE
5	36	F	08/08/2017	Sputum	*Citrobacter freundii* complex	ACH	Yes	None
6	75	M	09/01/2017	Urine	*Klebsiella pneumoniae*	ACH, LTACH	Unknown	None
7	56	F	11/28/2017	Urine	*Klebsiella oxytoca*	ACH	Yes	None

**Abbreviations:** ACH = acute care hospital; BAL = bronchoalveolar lavage; CDI = *Clostridioides difficile* infection; ESBL = extended-spectrum β-lactamase; F = female; LTACH = long term acute care hospital; LTCF = long term care facility; M = male; MDR-AB = multidrug-resistant *Acinetobacter baumannii* complex; MDR-PA = multidrug-resistant *Pseudomonas aeruginosa*; MRSA = methicillin-resistant *Staphylococcus aureus*; VRE = vancomycin-resistant *Enterococcus.*

* Single patient with multiple isolates.

Improved isolate submission and expanded capacity to detect carbapenemase-producing organisms have identified VIM-CRE as an emerging resistance problem in Indiana. All patients with VIM-CRE reported recent health care in Indiana but had not traveled outside the country, suggesting VIM transmission within Indiana health care facilities. Notably, although VIM remains one of the least frequently reported carbapenemases among CRE in the United States, Indiana and neighboring states account for 29 (71%) of the 41 VIM-CRE reported to CDC to date, suggesting possible regional emergence of this resistance mechanism (*6*). This finding highlights the important role of state public health laboratories in facilitating identification and reporting of CRE by clinical laboratories and in testing isolates to identify important CRE resistance mechanisms, including all five carbapenemases of major public health concern.* Although such testing has had limited availability in clinical and public health laboratories, recent CDC investments to create the Antibiotic Resistance Laboratory Network have increased carbapenemase testing and CRE screening nationwide. This testing will provide better understanding of CP-CRE epidemiology throughout the United States, including important regional differences in emerging carbapenemases (*6*). Once CP-CRE are identified, health department epidemiologists can work to ensure prompt implementation of infection control interventions. This collaboration between epidemiologists and laboratorians to identify, describe, and respond to emerging drug resistance is needed for containment efforts.

## Footnotes

* *Klebsiella pneumoniae* carbapenemase (KPC), New Delhi metallo-beta-lactamase (NDM), Verona integron-mediated metallo-β-lactamase (VIM), imipenemase (IMP), and oxacillinase-48-like carbapenemase (OXA-48).
